# Antimicrobial stewardship in high-risk febrile neutropenia patients

**DOI:** 10.1186/s13756-022-01084-0

**Published:** 2022-03-26

**Authors:** Adrien Contejean, Salam Abbara, Ryme Chentouh, Sophie Alviset, Eric Grignano, Nabil Gastli, Anne Casetta, Lise Willems, Etienne Canouï, Caroline Charlier, Frédéric Pène, Julien Charpentier, Jeanne Reboul-Marty, Rui Batista, Didier Bouscary, Solen Kernéis

**Affiliations:** 1 Faculté de Médecine, Université de Paris, 75006 Paris, France; 2 Service d’hématologie, AP-HP, APHP.CUP, Hôpital Cochin, 27 rue du Faubourg Saint-Jacques, 75014 Paris, France; 3 Équipe Mobile d’Infectiologie, AP-HP, APHP.CUP, Hôpital Cochin, 75014 Paris, France; 4 UVSQ, Inserm, CESP, Anti-infective Evasion and Pharmacoepidemiology Team, Université Paris-Saclay, 78180 Montigny-le-Bretonneux, France; 5 Institut Pasteur, Epidemiology and Modelling of Antibiotic Evasion (EMAE), 75015 Paris, France; 6 Laboratoire de bactériologie, AP-HP, APHP.CUP, Hôpital Cochin, 75014 Paris, France; 7 Equipe opérationnelle d’hygiène hospitalière, AP-HP, Hôpital Cochin, 75014 Paris, France; 8 Institut Pasteur, Biology of Infection Unit, INSERM U1117, French National Reference Center and WHO Collaborating Center Listeria, Paris, France; 9 Service de médecine intensive réanimation, AP-HP, APHP.CUP, Hôpital Cochin, 75014 Paris, France; 10 Département d’information médicale, AP-HP, APHP.CUP, Hôpital Cochin, 75014 Paris, France; 11 Pharmacie hospitalière, AP-HP, APHP.CUP, Hôpital Cochin, 75014 Paris, France; 12 INSERM, IAME, Université de Paris, 75006 Paris, France

**Keywords:** Antimicrobial stewardship, High-risk febrile neutropenia, Prognosis, Antibiotic consumption

## Abstract

**Background:**

The 2011 4th European Conference on Infections in Leukemia (ECIL4) guidelines recommend antibiotics de-escalation/discontinuation in selected febrile neutropenia (FN) patients. We aimed to assess the impact of an antimicrobial stewardship (AMS) program based on these guidelines on antibiotics use and clinical outcomes in high-risk FN patients.

**Methods:**

We conducted an observational study in the hematology department of Cochin University Hospital in Paris, France. An ECIL4-based antibiotics de-escalation and discontinuation strategy was implemented jointly by the hematologists and the AMS team. The pre-intervention (January–October 2018) and post-intervention (January-October 2019) periods were compared. We retrospectively collected clinical and microbiological data. We compiled antibiotics consumptions via hospital pharmacy data and standardized them by calculating defined daily doses per 1000 patient-days. We analyzed the two-monthly antibiotic consumption using an interrupted time series method and built a composite endpoint for clinical outcomes based on transfer to the intensive care unit (ICU) and/or hospital death.

**Results:**

Overall, 273 hospital stays (164 patients) in the pre-intervention and 217 (148 patients) in the post-intervention periods were analyzed. Patients were mainly hospitalized for intensive chemotherapy for acute leukemia or autologous stem-cell transplant for myeloma. Patients were slightly younger in the pre-intervention compared to the post-intervention period (median age 60.4 vs 65.2 years, p = 0.049), but otherwise comparable. After implementation of the AMS program, glycopeptide and carbapenem use decreased by 85% (p = 0.03) and 72% (p = 0.04), respectively. After adjustment on confounders, the risk of transfer to the ICU/death decreased significantly after implementation of the AMS program (post-intervention period: odds-ratio = 0.29, 95% Confidence Interval: 0.15–0.53, p < 0.001).

**Conclusion:**

Implementation of a multidisciplinary AMS program for high-risk neutropenic patients was associated with lower carbapenem and glycopeptide use and improved clinical outcomes.

**Supplementary Information:**

The online version contains supplementary material available at 10.1186/s13756-022-01084-0.

## Introduction

Febrile neutropenia (FN) is a potentially life-threatening complication occurring in patients with an absolute neutrophil count (ANC) below 0.5 × 10^9^/L [1]. Incidence of FN reaches 85–95% in patients receiving intensive chemotherapy for acute myeloid leukemia (AML) [2] and depends mainly on the depth and duration of neutropenia [3]. Broad-spectrum antibiotics use is the cornerstone of initial FN management and is guided by the 2011 4^th^ European Conference on Infections in Leukemia (ECIL4). These guidelines recommended to discontinue antibiotics in patients with non-severe fever of unknown origin (FUO) after three days and 48 h of apyrexia regardless of the ANC. They also proposed a de-escalation strategy in selected patients [1]. These recommendations have been recently reinforced by a randomized controlled trial showing that stopping antibiotics after three days of apyrexia in patients with FUO was safe and even beneficial in high-risk neutropenic patients without antibacterial prophylaxis [4]. Strict application of ECIL4 guidelines on de-escalation and discontinuation strategies seems finally feasible, safe and would decrease broad-spectrum antibiotic use [5]. Despite these high-level recommendations, de-escalation strategies (i.e., to narrower-spectrum β-lactams or discontinuation of antibiotics in patients with no evidence of infection) are still the subject of intense debates among hematologists and infectious diseases specialists and remain rarely applied in neutropenic patients.

In this longitudinal study, we evaluated, in real-life conditions, the impact on antibiotic use and clinical outcomes of an antimicrobial stewardship (AMS) program based on ECIL4 guidelines in high-risk neutropenic patients, in a hematologic intensive care ward in Paris, France.

## Methods

### Setting

The present study was conducted in the Hematology department of the Cochin University Hospital in Paris, France (24 beds of hematology in a 1074 beds tertiary-care university center). The center takes care of adults with all types of hematological diseases and performs autologous stem-cell transplant, but not allogeneic transplant. The department is divided in two 12-beds distinct units: a hematologic intensive care ward where patients mainly receive intensive chemotherapy and autologous stem-cell transplant, and a conventional hospitalization unit. No antibacterial prophylaxis was routinely provided to patients. Antifungal prophylaxis was administered according to ECIL guidelines [6].

### Antimicrobial stewardship policy

The local guidelines for management of high-risk neutropenic patients were elaborated in November–December 2018 and jointly endorsed by the AMS, hematology and intensive care unit (ICU) senior physicians. They were presented in a joint meeting including all staff of both AMS and hematology teams. The decisional chart was displayed in all medical offices and the detailed protocol was made available in the local network reachable via the hospital’s computers. During the study time, the AMS team was composed of three senior physicians and one resident. One senior physician in the hematology team was identified as the priority correspondent for infectious problems and discussions with the AMS team. The daily weekday routine for the whole hospital included a systematic consultation and physical examination by a member of the team for every patient with a positive blood culture, associated with on-demand phone counseling or specific consultations for other patients. All medical charts were reviewed twice a week by the AMS team and the hematologists during dedicated meetings, to make decisions on difficult cases and promote the guidelines. Annual feedbacks on antibiotic consumption in hematology were set-up. New AMS and hematology residents were systematically trained to these new guidelines every 6 months.

### Local high-risk febrile neutropenia guidelines

Local guidelines were based on ECIL4 recommendations, as detailed in Additional file 1: Appendix [1]. High-risk patients with FN were defined as patients with ANC below 0.5 × 10^9^/L for an anticipated duration of more than 7 days, with a body temperature ≥ 38.3 °C once or ≥ 38 °C on two occasions, at least one hour apart. All patients with high-risk FN had catheter and peripheral blood cultures and other samplings (such as sputum or urine) guided by clinical examination. First-line therapy relied on piperacillin/tazobactam (or meropenem in patients with risk factors for extended spectrum *β*-lactamase producing *Enterobacteriaceae* [ESBL-PE] infection, for further detail see the Additional file 1: Appendix). Amikacin was additionally administered in patients with severity criteria, while vancomycin was added in those with high suspicion of infection with a resistant gram-positive microorganism. Antibiotic treatments were systematically reassessed at 48–72 h in collaboration with the AMS team. In accordance with ECIL4 guidelines, persistence or reoccurrence of fever without clinical deterioration or new clinical symptoms did not lead to antibiotic change, except in patients colonized with ESBL-PE. Patients with microbiological documentation and apyrexia were eligible for de-escalation targeting the documented infection. Patients on meropenem without initial severity criteria or microbiological documentation were eligible for de-escalation to piperacillin/tazobactam. In patients with FUO, antibiotics could be stopped after 3 days of treatment and 48 h of apyrexia. In patients with clinically or microbiologically documented infection, antibiotics were continued until day 7 and 4 days of apyrexia. For patients with fever recurrence after antibiotics discontinuation, antibiotics were resumed, and the protocol restarted at its beginning. Patients with initial severity criteria, receiving corticosteroids, or with complex infections, were excluded from these guidelines and received tailored-fit antibiotic treatment.

### Patients

In this study, we compared two periods: the pre-intervention period from January to October 2018, before implementation of these guidelines, and the post-intervention period from January to October 2019, after implementation. All patients hospitalized in the intensive hematology ward during one of these periods were eligible and included in the analysis.

Local ICU admission policies did not change between the two periods. Patients were transferred to the ICU after clinical evaluation from both hematology and ICU physicians for close cardiovascular, respiratory or neurological monitoring, or in case of organ failure.

### Data

Patients’ age, sex, comorbidities, hematological malignancy, dates of stay, cause of hospitalization, hospital stay complications, ICU transfer, and cause of death were retrospectively collected from the biomedical informatics department data. Dates and lengths of stays were censored on January 1st and October 31st for each period.

Microbiological data were retrieved from the microbiological department software GLIMS (version 8.11.14, CliniSys Group, Gent, Belgium). All *Pseudomonas aeruginosa* and ESBL-PE infections were reviewed using individual patients’ file by a clinical investigator (AC).

Antibiotic consumption was calculated using hospital pharmacy data. For each period of two months, the pharmacy calculated the balance between the antibiotics delivered to the hematologic intensive care ward and those returned to the pharmacy. We standardized the antibiotic consumption by calculating the defined daily dose (DDD) per 1000 patient-days for each two-months step [7]. Antibiotics were classified using a consensual ranking of β-lactams, class 3 *β*-lactams corresponding to third-generation cephalosporins or ureido/carboxy-penicillins, and class 4 *β*-lactams corresponding to piperacillin/tazobactam, fourth-generation cephalosporins and antipseudomonal third-generation cephalosporins [8]. The consumption of daptomycin and linezolid were pooled for the analyses since their use were marginal in our unit and considered as alternatives to glycopeptide in the present study.

We retrospectively used ICU data to evaluate the number of stays in the ICU for each period. The overall ICU transfer rate and the number of ICU transfers of more than 24 h were calculated (to avoid short stays in the ICU for medical procedures like central catheter insertion or scheduled hemodialysis).

### Statistical methods

Continuous variables are presented as median [interquartile range] and categorical variables as number (percentage). Fisher exact tests were used for comparisons of qualitative variables and Mann–Whitney tests for quantitative variables. All tests were 2-sided with a 0.05 value for significance.

We estimated the two-monthly consumption of antibiotics with a multiple linear regression model

$${\text{consumption}}_{t} = \left\{ {\begin{array}{*{20}l} {\beta_{0} + \beta_{1} \times t,} \hfill & {{\text{pre - intervention}}\,{\text{period}}} \hfill \\ {\beta_{0} + (\beta_{1} + \beta_{2} ) \times t,} \hfill & {{\text{ post - intervention}}\,{\text{period}}} \hfill \\ \end{array} } \right.$$

In this model, *t* is a continuous variable indicating the time in bimester since 2018. *β*_*0*_ is the intercept which estimates the baseline consumption of the evaluated antibiotic, *β*_*1*_ is the change in consumption during the pre-intervention period, and *β*_*2*_ is the change of slope between the pre-intervention period and the post-intervention period.

We used a logistic regression model to assess the risk factors associated with a composite endpoint: ICU transfer for more than 24 h or hospital death. The following variables were included in the model: age > 60 years, female sex, Charlson comorbidity index > 3 [9], acute myeloid leukemia, hospitalization for intensive or induction chemotherapy, hospital stay with at least one febrile episode, and post-intervention period of hospitalization. Survival curves were built following the Kaplan–Meier method and statistical comparison with the log-rank test.

Statistical analyses were performed using R-software (3.3.2, R Foundation for Statistical Computing, Vienna, Austria).

## Results

During the study period, 490 hospital stays were analyzed, including 273 during the pre-intervention period (n = 164 unique patients) and 217 during the post-intervention period (n = 148 unique patients). As shown in Table 1, patients were slightly younger in the pre-intervention period (age: 60.4 [49.4–71.9] versus 65.2 [54.3–72.8] years, respectively, p = 0.049), but the sex ratio, Charlson comorbidity index and underlying hematological diseases were not different. The total number of patient-days was similar in the two periods (3180 and 3129 in the pre- and post-intervention periods, respectively). The most represented hematological malignancies were myeloma, AML and aggressive lymphoma, accounting for more than half of the patients. Causes of hospitalization were equally distributed between periods. Patients were hospitalized mainly for intensive or induction chemotherapy or consolidation chemotherapy for AML or acute lymphoblastic leukemia. About one in five patients was admitted for autologous stem-cell transplant for myeloma or lymphoma. The number of stays with at least one febrile episode was lower during the pre-intervention period (118/273 [43.2%]) than the post-intervention period (116/217 [53.5%], p = 0.031).

**Table 1:** Overall population characteristics (per patients) and comparison of hospital stays between pre- and post-intervention periods

Patients characteristics	Pre-intervention period Jan–Oct 2018 N = 164 patients	Post-intervention period Jan–Oct 2019 N = 148 patients	p value
Age (year); Median [IQR]	60.4 [49.4–71.9]	65.2 [54.3–72.8]	0.049
Sex (female); N (%)	78 (47.6)	65 (43.9)	0.60
Charlson comorbidity index; Median [IQR]	2 [2–4]	2 [2–6]	0.54
Number of stays; Median [IQR]	1 [1–2]	1 [1–2]	0.49
Hematological disease; N (%)			0.44
Myeloma	37 (22.6)	45 (30.4)	
Acute myeloid leukemia	31 (18.9)	32 (21.6)	
Aggressive lymphoma	28 (17.1)	26 (17.6)	
Indolent lymphoma	20 (12.2)	9 (6.1)	
ALL/LBL	18 (11)	11 (7.4)	
Myelodysplastic syndrome	7 (4.3)	9 (6.1)	
Hodgkin lymphoma	6 (3.7)	4 (2.7)	
Aplastic anemia	5 (3)	5 (3.4)	
Other	12 (7.3)	7 (4.7)	

Hospital stays characteristics	Pre-intervention period Number of hospital stay = 273	Post-intervention period Number of hospital stay = 217	p-value
Total number of patient-days	3180	3129	–
Cause of hospitalization; N (%)			0.28
Intensive or induction chemotherapy	57 (20.9)	38 (17.5)	
Leukemia consolidation chemotherapy	32 (11.7)	27 (12.4)	
Chemotherapy (other)	67 (24.5)	49 (22.6)	
Autologous BMT	51 (18.7)	48 (22.1)	
Transfusion	9 (3.3)	9 (4.1)	
Palliative care	4 (1.5)	5 (2.4)	
Aplasia	1 (0.4)	5 (2.4)	
Antithymocyte globulin + ciclosporin	1 (0.4)	4 (1.8)	
Other	51 (18.6)	32 (14.7)	
Number of stays with febrile episode; N (%)	118 (43.2)	116 (53.5)	0.031

ALL: Acute lymphoblastic leukemia; LBL: Lymphoblastic lymphoma

**Fig. 1:**
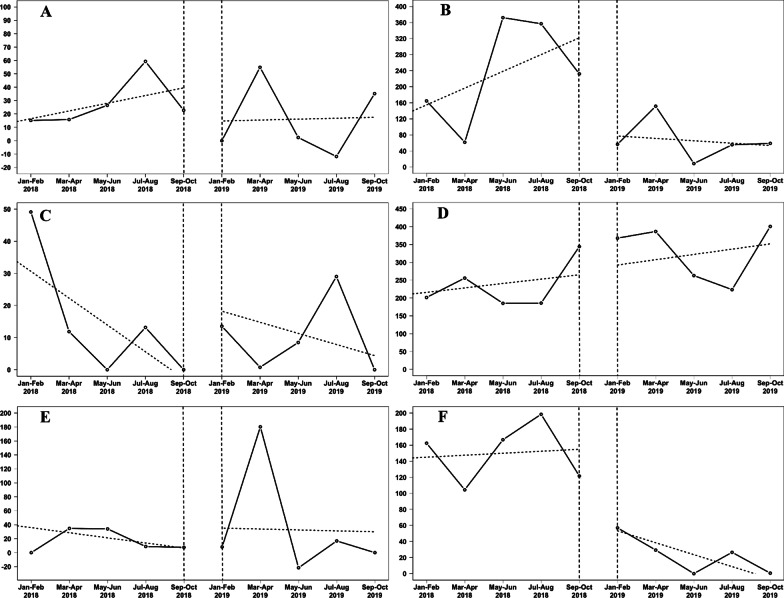
Segmented linear regression of antibiotics consumption during study period in defined daily dose per 1000 patient-days over time. **A** Aminoglycosides; **B** Carbapenems; **C** Class 3 β-lactams (third-generation cephalosporins or ureido/carboxy-penicillins); **D** Class 4 β-lactams (piperacillin/tazobactam, fourth-generation cephalosporins and antipseudomonal third-generation cephalosporins), **E** Fluoroquinolones; **F** Glycopeptides. Solid lines represent the crude 2-months antibiotic consumption. The dashed lines represent the linear regression for each period

Unadjusted raw glycopeptide consumption sharply dropped by 85%, from 754 DDD/1000 patient-days in the pre-intervention period to 113 DDD/1000 patient-days in the post-intervention period (Table 2). Moreover, raw carbapenem consumption decreased by 72%, from 1187 DDD/1000 patient-days in the pre-intervention period to 331 DDD/1000 patient-days in the post-intervention period. Interrupted time series analysis showed a significant change of slope (Table 2, Fig. 1) in the post-intervention period for both glycopeptides and carbapenems consumption. Consumption of aminoglycosides, class 3 or 4 β-lactams, fluoroquinolones, daptomycin/linezolid and cotrimoxazole did not statistically change between the two study periods (Table 2, Fig. 1).

**Table 2:** Antibiotic use over time: total consumption and parameters of the multiple linear regression model

Antibiotics	Intercept *β*_0_ [CI]	Period 1			Period 2		
		Total	*β*_1_ [CI]	p-value	Total	*β*_2_ [CI]	p-value
Aminoglycosides	+ 10.69 [− 44.67; + 66.05]	140	+ 5.77 [− 11.18; + 22.71]	0.45	81	− 5.09 [− 16.90; + 6.71]	0.34
Glycopeptides	+ 142.54 [+ 71.56; + 213.51]	754	+ 2.47 [− 19.26; + 24.19]	0.80	113	− 17.34 [− 32.48;− 2.21]	0.03
Carbapenems	+ 112.95 [− 100.10; + 325.99]	1187	+ 41.78 [− 23.42; + 106.99]	0.17	331	− 47.73 [− 93.15;− 2.31]	0.04
Class 3 *β*-lactams	+ 39.00 [+ 6.48; + 71.51]	74	− 8.35 [− 18.30; + 1.60]	0.09	52	+ 4.89 [− 2.05; + 11.82]	0.14
Class 4 *β*-lactams	+ 203.79 [+ 18.64; + 388.94]	1173	+ 12.32 [− 44.34; + 68.98]	0.62	1642	+ 2.49 [− 36.98; + 41.96]	0.89
Fluoroquinolones	+ 43.18 [− 101.31; + 187.68]	85	− 7.32 [− 51.55; + 36.90]	0.71	183	+ 5.99 [− 24.81; + 36.80]	0.66
Daptomycine + Linezolide	+ 6.43 [− 33.54; + 46.40]	56	+ 2.01 [− 10.22; + 14.25]	0.71	37	− 2.05 [− 10.57; + 6.47]	0.59
Cotrimoxazole	+ 32.75 [− 20.97; + 86.46]	286	+ 7.70 [− 8.74; + 24.14]	0.30	86	− 9.48 [− 20.93; + 1.97]	0.09
All antibiotics	+ 1692.82 [+ 1235.32; + 2150.33]	6848	− 105.66 [− 245.67; + 34.36]	0.12	3473	− 19.91 [− 117.45; + 77.63]	0.64

Total antibiotic consumption is expressed in defined daily dose per 1000 patient-days. Raw consumption of daptomycin was 35 DDD/1000 patient-days in both pre- and post-intervention periods. Raw consumption of linezolide was 21 DDD/1000 patient-days in the pre-intervention period and 2 DDD/1000 patient-days in the post-intervention period

CI: 95% confidence interval; Class 3 *β*-lactams: third-generation cephalosporins or ureido/carboxy-penicillins; Class 4 *β*-lactams: piperacillin/tazobactam, fourth-generation cephalosporins or antipseudomonal third-generation cephalosporins

**Fig. 2:**
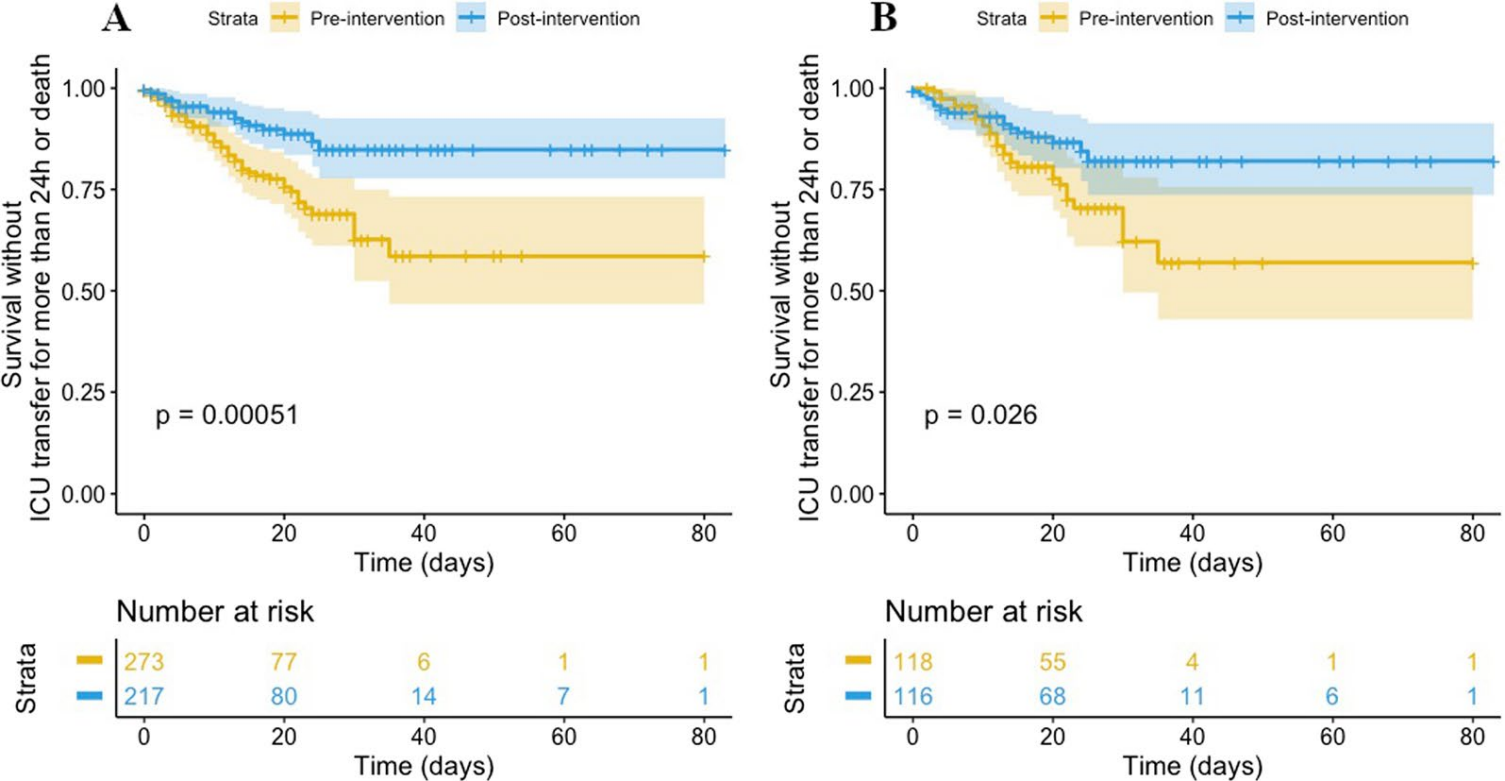
Kaplan–Meier curves for occurrence of a negative outcome (ICU transfer for more than 24 h or death). A: Overall study population; B: Hospital stays with at least one febrile episode. Log-rank tests were used for statistical comparisons. Faded areas represent the 95% confident interval of each curve

Length of stay was not statistically different between groups (median of 9 [3–19] days in the pre-intervention period versus 13 [3–21] days in the post-intervention period, p = 0.061). During the pre-intervention period as compared with the post-intervention period, there were significantly more stays with transfer to the ICU for more than 24 h, and more stays with the composite endpoint *i.e.* ICU transfer for more than 24 h or hospital death (49/273, 17.4%, versus 18/217, 8.3%, p = 0.0020, Fig. 2A, Table 3). The result on the composite endpoint was similar after restriction to the subgroup of hospital stays marked by at least one febrile episode (Fig. 2B, Table 3). We found no difference in the hospital death rate between the two periods (14/273, 5.1%, versus 8/217, 3.7%, in the pre- and post-intervention periods, respectively, p = 0.59) (Table 3). The logistic regression model (Table 4) identified age > 60 years (odds-ratio (OR) = 2.14, 95% confidence interval, [1.22–3.85]), Charlson comorbidity index > 3 (OR = 3.17 [1.80–5.69]), hospital stay with fever (OR = 3.10 [1.75–5.67]), and the post-intervention period (OR = 0.29 [0.15–0.53]) as being independently associated with the composite clinical endpoint (ICU transfer for > 24 h or hospital death). Four patients experienced a *Clostridioides difficile* colitis in the pre-intervention period and six in the post-intervention period. In the pre-intervention period, 4/5 *Pseudomonas aeruginosa* infections were related to a meropenem-resistant strain, compared to 2/8 in the post-intervention period (p = 0.10). Invasive infections with ESBL-PE emerged in 9/273 (3.3%) stays in the pre-intervention period versus 2/217 (0.9%) stays in the post-intervention period (p = 0.12).

**Table 3:** Outcomes

Outcome	Pre-intervention period Number of hospital stays = 273	Post-intervention period Number of hospital stays = 217	p-value
Length of stay (days); Median [IQR]	9 [3–19]	13 [3–21]	0.061
*Clostridioides difficile* infections; N (%)	4 (1.5)	6 (2.8)	0.35
Invasive infection with ESBL-PE; N (%)	9 (3.3)	2 (0.9)	0.12
Meropenem-resistant isolates among *Pseudomonas aeruginosa* infections; N (%)	4/5 (80)	2/8 (25)	0.10
Transfer to the ICU for more than 24 h or death; N (%)	49 (17.9)	18 (8.3)	0.0020
Transfer to the ICU for more than 24 h; N (%)	42 (15.4)	10 (4.6)	0.00022
Death; N (%)	14 (5.1)	8 (3.7)	0.59

Comparison of outcomes in the pre- and post-intervention periods in terms of length of stay, infectious complications and ICU transfer or death

ICU: Intensive Care Unit

**Table 4:** Logistic regression model: risk factors associated with the composite endpoint (transfer to ICU for more than 24 h or hospital death)

Variables	Univariate analysis		Multivariate analysis	
	OR [95% CI]	p-value	OR [95% CI]	p-value
Sex				
Female	0.77 [0.45–1.29]	0.33	0.78 [0.44–1.35]	0.37
Male	Ref		Ref	
Age				
> 60 years	1.80 [1.06–2.11]	0.031	2.14 [1.22–3.85]	0.0093
≤ 60 years	Ref		Ref	
Charlson comorbidity index				
> 3	2.59 [1.54–4.39]	< 0.001	3.17 [1.80–5.69]	< 0.001
≤ 3	Ref		Ref	
Hematological disease				
Acute myeloid leukemia	1.37 [0.75–2.40]	0.29	1.67 [0.82–3.34]	0.15
Other	Ref		Ref	
Cause of hospitalization				
Intensive or induction chemotherapy	1.12 [0.57–2.06]	0.74	0.99 [0.45–2.08]	0.99
Other	Ref		Ref	
At least one febrile episode during hospital stay				
Yes	2.53 [1.48–4.44]	< 0.001	3.10 [1.75–5.67]	< 0.001
No	Ref		Ref	
Period				
Post-intervention period	0.41 [0.23–0.72]	0.0025	0.29 [0.15–0.53]	< 0.001
Pre-intervention period	Ref		Ref	

Results of both univariate and multivariate analyses are presented

OR: Odds-ratio; ref: reference

## Discussion

At the scale of an intensive hematology unit, implementation of a multidisciplinary AMS program for high-risk febrile neutropenia patients was feasible and well accepted, was associated with a dramatic decrease in the carbapenem and glycopeptide consumptions and with a better outcome for the patients.

The decrease in carbapenem and glycopeptide consumption was substantial after implementation of our local guidelines and was not associated with an increase in daptomycin/linezolid consumption. Consumption of class 4 β-lactams remained relatively stable, which could reflect the combined effects of class 4 β-lactams discontinuation for some patients, de-escalation from a carbapenem to class 4 β-lactams in some situations, and the absence of escalation to a carbapenem in others. Interestingly, we noted a trend towards lower rates of both ESBL-PE and meropenem-resistant *Pseudomonas aeruginosa* invasive strains, but numbers were too small to reach statistical significance. There was no difference in the incidence of *Clostridioides difficile* infections. Indeed, *Clostridioides difficile* infections are more strongly associated with the use of 4C antibiotics (fluoroquinolones, clindamycin, co-amoxiclav, and cephalosporins) than with carbapenem use [10]. Carbapenems are very broad-spectrum β-lactams efficient against ESBL-PE and anaerobic bacteria. Although data are scarce and contentious [11], it is likely that these drugs are associated with alterations of the fecal microbiota and promote the fecal carriage of carbapenem-resistant Gram-negative bacilli [12]. Previously published studies have suggested that prior exposition to carbapenem was strongly associated with emergence of carbapenem-resistance in *Pseudomonas aeruginosa* [12]. Moreover, anti-anaerobic antibiotics such as carbapenems (but also piperacillin/tazobactam) or vancomycin promote high-density colonization with vancomycin-resistant enterococci (VRE) in patients’ stool [13]. In this regard, the implementation of AMS programs in hematology, by decreasing carbapenem and glycopeptide consumption, may have an impact on VRE infections [14]. Furthermore, reducing antibiotic consumption in patients with FN has previously been reported as cost-saving [5]. Finally, vancomycin, the most used glycopeptide in patients with febrile neutropenia [15], is known to be associated with side effects such as acute kidney injury by direct renal toxicity or secondary to a drug-drug interaction with piperacillin [16, 17]. All these data suggest that decreasing antibiotic exposure, especially to carbapenems and glycopeptides, may be beneficial for FN patients in multiple ways.

We observed a significant drop of ICU transfers in the post-intervention period. In this respect, the implementation of our AMS program was safe and even probably beneficial by contributing to an improvement in the overall prognosis of the patients. Such large difference was unexpected since previous AMS intervention studies, which were however designed differently, were not associated with such a strong improvement of prognosis [5, 14]. However, the Charlson comorbidity index, cause of hospitalization, hematological diseases of patients were similar between the two periods, and all analyses were adjusted on these variables. Moreover, our results are in line with those of Aguilar-Guisado et al., who showed that early discontinuation of antibiotics in patients with FUO was associated with less serious adverse events than continuation of antibiotics until ANC recovery [4]. Decreasing exposure to carbapenem in high-risk febrile neutropenia patients likely reduces selective pressure and contributes to preventing subsequent colonization/infection with multi-drug resistant bacteria. Discontinuing parenteral antibiotics also decreases the risks of catheter-related infections, other healthcare-associated infections and antibiotics side effects. Lastly, our results might also reflect a more careful surveillance and better diagnostic work-up in patients after discontinuation/de-escalation of antibiotics. Tremendous implication of both the hematological medical, paramedical and AMS teams to ensure that the patient receives the best care may have contributed to improving general care and outcomes, beyond infectious considerations.

AMS programs have been shown to reduce antibiotic use, hospital costs, incidence of infection and colonization with antibiotic-resistant bacteria and *Clostridioides difficile* infections in various populations of patients, and are key to curtail antimicrobial resistance [18]. Our study population reflects the typical population of an intensive hematology unit. Most patients were undergoing intensive chemotherapy or autologous stem-cell transplant, both situations being at high-risk of FN as exemplified by the rate of stays with at least one febrile episode of 48% in the entire cohort. In this specific population, AMS programs have been recently implemented in several hematology units, with very encouraging results [5, 14, 19]. Published data suggest that AMS interventions in hematology are safe and well accepted by clinicians, but knowledge gaps persist regarding antibiotics de-escalation and discontinuation strategies in high-risk FN patients [15, 18]. Broad development and evaluation of multidisciplinary AMS programs in this population of patients is thus warranted [20]. In our study, the specific organization in the post-intervention period with identification of a priority correspondent for infectious diseases discussions and systematic scheduled meetings may have been key for the success of the intervention [15, 19].

Several limitations must be acknowledged. First, the retrospective design of this study did not allow to evaluate each patient encounters individually and evaluate all potential confounding factors. For example, “induction chemotherapy for AML” may gather various type of chemotherapies with different toxicity profiles (such as daunorubicin/cytarabine, CPX-351 liposome or 5-azacytidin/venetoclax), however the statistical impact of this heterogeneity should be marginal given the sample size. Likewise, we could not precisely assess patients’ severity or calculate their performance status. Still, age, comorbidities, Charlson comorbidity index and cause of hospitalization were included in the multivariate logistic regression model. Second, we were not able to include patients with allogeneic stem-cell transplant, which limits the external value of our results, but our population was otherwise highly representative of high-risk hematology patients including patients with acute leukemia, lymphoma and myeloma undergoing intensive chemotherapy or autologous stem-cell transplant. Last, using non-standard doses of antibiotics can affect calculation of DDD [21]. However, local practices regarding antibiotics doses were not concerned by the implemented guidelines, thus antibiotics dosing regimen did not change during study time.

## Conclusion

Implementation of a de-escalation and discontinuation strategy based on ECIL4 guidelines for patients with high-risk FN in our center was feasible, safe, and led to a significant decrease in glycopeptide and carbapenem consumption at the scale of an intensive hematology unit. The overall standard of care was impacted, with significantly less ICU transfers after the intervention. Also, it was accompanied by a trend towards fewer meropenem-resistant *Pseudomonas aeruginosa* infections, although larger studies are warranted to confirm this observation. A multidisciplinary approach, with endorsement of guidelines by both hematology and AMS teams, and close collaboration for patient care appear to be key factors in the success of such programs in this specific population.

## Supplementary Information

**Additional file 1:** Local high-risk febrile neutropenia guidelines.

## Data Availability

The datasets used and/or analyzed during the current study are available from the corresponding author on reasonable request.

## References

[1] Averbuch D, Orasch C, Cordonnier C, Livermore DM, Mikulska M, Viscoli C (2013). European guidelines for empirical antibacterial therapy for febrile neutropenic patients in the era of growing resistance: summary of the 2011 4th European Conference on Infections in Leukemia. Haematologica.

[2] Taplitz RA, Kennedy EB, Bow EJ, Crews J, Gleason C, Hawley DK (2018). Antimicrobial prophylaxis for adult patients with cancer-related immunosuppression: ASCO and IDSA clinical practice guideline update. J Clin Oncol.

[3] Bodey GP, Buckley M, Sathe YS, Freireich EJ (1966). Quantitative relationships between circulating leukocytes and infection in patients with acute leukemia. Ann Intern Med.

[4] Aguilar-Guisado M, Espigado I, Martín-Peña A, Gudiol C, Royo-Cebrecos C, Falantes J (2017). Optimisation of empirical antimicrobial therapy in patients with haematological malignancies and febrile neutropenia (How Long study): an open-label, randomised, controlled phase 4 trial. Lancet Haematol.

[5] la Martire G, Robin C, Oubaya N, Lepeule R, Beckerich F, Leclerc M (2018). De-escalation and discontinuation strategies in high-risk neutropenic patients: an interrupted time series analyses of antimicrobial consumption and impact on outcome. Eur J Clin Microbiol Infect Dis.

[6] Maertens JA, Girmenia C, Brüggemann RJ, Duarte RF, Kibbler CC, Ljungman P (2018). European guidelines for primary antifungal prophylaxis in adult haematology patients: summary of the updated recommendations from the European Conference on Infections in Leukaemia. J Antimicrob Chemother.

[7] Stanic Benic M, Milanic R, Monnier AA, Gyssens IC, Adriaenssens N, Versporten A (2018). Metrics for quantifying antibiotic use in the hospital setting: results from a systematic review and international multidisciplinary consensus procedure. J Antimicrob Chemother.

[8] Weiss E, Zahar J-R, Lesprit P, Ruppe E, Leone M, Chastre J (2015). Elaboration of a consensual definition of de-escalation allowing a ranking of β-lactams. Clin Microbiol Infect.

[9] Charlson ME, Pompei P, Ales KL, MacKenzie CR (1987). A new method of classifying prognostic comorbidity in longitudinal studies: development and validation. J Chronic Dis.

[10] Lawes T, Lopez-Lozano J-M, Nebot CA, Macartney G, Subbarao-Sharma R, Wares KD (2017). Effect of a national 4C antibiotic stewardship intervention on the clinical and molecular epidemiology of Clostridium difficile infections in a region of Scotland: a non-linear time-series analysis. Lancet Infect Dis.

[11] Woerther P-L, Lepeule R, Burdet C, Decousser J-W, Ruppé É, Barbier F (2018). Carbapenems and alternative β-lactams for the treatment of infections due to extended-spectrum β-lactamase-producing Enterobacteriaceae: what impact on intestinal colonisation resistance?. Int J Antimicrob Agents.

[12] Armand-Lefèvre L, Angebault C, Barbier F, Hamelet E, Defrance G, Ruppé E (2013). Emergence of imipenem-resistant gram-negative bacilli in intestinal flora of intensive care patients. Antimicrob Agents Chemother.

[13] Donskey CJ, Chowdhry TK, Hecker MT, Hoyen CK, Hanrahan JA, Hujer AM (2000). Effect of antibiotic therapy on the density of vancomycin-resistant enterococci in the stool of colonized patients. N Engl J Med.

[14] Webb BJ, Majers J, Healy R, Jones PB, Butler AM, Snow G (2020). Antimicrobial stewardship in a hematological malignancy unit: carbapenem reduction and decreased vancomycin-resistant enterococcus infection. Clin Infect Dis.

[15] So M (2019). Antimicrobial stewardship in patients with hematological malignancies: key considerations. Curr Treat Options Infect Dis.

[16] Contejean A, Tisseyre M, Canouï E, Treluyer J-M, Kerneis S, Chouchana L. Combination of vancomycin plus piperacillin and risk of acute kidney injury: a worldwide pharmacovigilance database analysis. J Antimicrob Chemother. 2021. 10.1093/jac/dkab003 33617641

[17] Bellos I, Karageorgiou V, Pergialiotis V, Perrea DN (2020). Acute kidney injury following the concurrent administration of antipseudomonal β-lactams and vancomycin: a network meta-analysis. Clin Microbiol Infect.

[18] Baur D, Gladstone BP, Burkert F, Carrara E, Foschi F, Döbele S (2017). Effect of antibiotic stewardship on the incidence of infection and colonisation with antibiotic-resistant bacteria and Clostridium difficile infection: a systematic review and meta-analysis. Lancet Infect Dis.

[19] So M, Mamdani MM, Morris AM, Lau TTY, Broady R, Deotare U (2018). Effect of an antimicrobial stewardship programme on antimicrobial utilisation and costs in patients with leukaemia: a retrospective controlled study. Clin Microbiol Infect.

[20] Gyssens IC, Kern WV, Livermore DM (2013). The role of antibiotic stewardship in limiting antibacterial resistance among hematology patients. Haematologica.

[21] Barlam TF, Cosgrove SE, Abbo LM, MacDougall C, Schuetz AN, Septimus EJ (2016). Implementing an antibiotic stewardship program: guidelines by the Infectious Diseases Society of America and the Society for Healthcare Epidemiology of America. Clin Infect Dis.

