# Supplementary material for: Antimicrobial stewardship in high-risk febrile neutropenia patients
Source: Antimicrob Resist Infect Control. 2022 Mar 26;11:52. doi: 10.1186/s13756-022-01084-0 (PMC8961889; doi:10.1186/s13756-022-01084-0)
Supplement: Supplementary file 1 — Additional file 1: Local high-risk febrile neutropenia guidelines. [file 13756_2022_1084_MOESM1_ESM.docx]

**Antimicrobial stewardship in high-risk febrile neutropenia patients**

**Supplementary appendix**

1. **Definition of high-risk febrile neutropenia**

- Absolute neutrophile count bellow 0.5x10^9^/L for an expected time of more than 7 days
- Body temperature ≥ 38.3°C once or ≥ 38°C twice one hour apart.

1. **Initial antibiotherapy**

**Addition of VANCOMYCIN in the following situations**

≥ 1 criterion for severity **AND** MRSA colonization

**OR** central catheter suspect of infection

**OR** Skin and soft tissue infection

*Criteria for severity

- Systolic blood pressure < 90mmHg or mottling or arterial lactate level ≥ 2mmol/L
- Oxygen saturation < 92% in room air or respiratory rate ≥ 22/min or breathing difficulties
- Disorders of consciousness

** Broad spectrum antibiotics: quinolones, 3rd generation cephalosporin, piperacillin/tazobactam

ESBL-E: Extended-spectrum β-lactamase Producing *Enterobacteriaceae*; MRSA: Methicillin-resistant *Staphylococcus aureus* ; PIP/TAZ: Piperacillin/Tazobactam

*In case of penicillin allergy:*

- *With no contraindication to cephalosporins: cefepime 2g every 8h or ceftazidime 2g over 30 minutes followed by 6g/24h by continuous infusion.*
- *Contraindication to cephalosporins and no severity: aztreonam 2g every 8h + vancomycin*
- *Contraindication to cephalosporins and severity: meropenem 2g every 8h.*

1. **Reassessment at 48-72h**

Persistence or reoccurrence of fever without clinical deterioration or new clinical symptom is not an indication to modify antibiotics, except in patients colonized with Extended-spectrum β-lactamase Producing *Enterobacteriacea* (ESBL-E).

PIP/TAZ: Piperacillin/Tazobactam

1. **Duration of treatment after stabilization**
